# Nursing students’ perceptions of image-based anatomical self-assessment in undergraduate education

**DOI:** 10.3389/fmed.2026.1826706

**Published:** 2026-05-25

**Authors:** Rafael Martínez-Gómez, Arturo Garcia-Garcia, José Vicente Carmona-Simarro, Marta Pérez-Doménech, Elena Fortes-Montoya, Esther Ferrer-Siscar, Noelia Rodríguez-Blanco, David Alarcón-Alarcón, Martín Pérez-Leal

**Affiliations:** 1 Faculty of Health Science, Universidad Europea de Valencia, Valencia, Spain; 2 Hospital Emergency Department, Hospital University Vinalopó, Elche, Spain; 3 Centro Avanzado de Microbiología Aplicada (CAMA), Universitat Politècnica de Valencia, Valencia, Spain

**Keywords:** anatomy, computer-assisted instruction, education, nursing, educational measurement, self-assessment

## Abstract

**Background:**

Human anatomy is a core subject in undergraduate nursing education but is often perceived as challenging due to its conceptual complexity and visual–spatial demands. Digital self-assessment tools may support active learning and reinforce anatomical identification through repeated engagement with visual content.

**Objective:**

To evaluate first-year nursing students’ perceptions of the educational value, acceptability, and satisfaction associated with an image-based anatomy self-assessment activity designed to reinforce anatomical identification across the full syllabus under real educational conditions.

**Methods:**

A cross-sectional descriptive study was conducted among first-year nursing students enrolled in a Human Anatomy module. The activity comprised 266 image-based identification and ordering questions delivered online as part of continuous assessment. Student perceptions were collected through a post-activity questionnaire with a Net Promoter Score (NPS) item and an open-ended question. Demographic and academic variables were recorded. Analyses included descriptive statistics, non-parametric tests, and k-means clustering; qualitative comments were examined through content-based analysis.

**Results:**

A total of 159 students completed the activity and questionnaire (93.0%). Satisfaction was high (mean NPS 7.97; NPS index +27), with 49.1% classified as Promoters. Performance scores showed a ceiling effect (median 8.8/10; 98.1% pass rate). No significant differences in satisfaction or performance were observed across demographic or academic subgroups. Qualitative feedback highlighted perceived utility as the most frequent theme, with concerns focused on image quality, technical issues, and workload. Cluster analysis identified three student profiles, indicating that satisfaction varied mainly by experiential rather than demographic factors.

**Conclusion:**

The image-based self-assessment activity was well accepted and perceived as educationally valuable, supporting anatomical identification across student subgroups. Improving image clarity, technical stability, and workload may further enhance learning. Findings support integrating structured image-based self-assessment as a complementary tool in nursing anatomy education.

## 1 Introduction

Human anatomy is a cornerstone of health science curricula, providing essential clinical skills such as physical examination, diagnostic imaging interpretation, and procedural performance (1). Despite its recognized importance, anatomy is frequently perceived by students as one of the most difficult subjects to master (2). This perception is largely attributable to the extensive volume and complexity of anatomical information, the high cognitive load imposed on novice learners, and the requirement to mentally visualize three-dimensional structures (3). Limited prior exposure to anatomy further compounds these difficulties, often leading students to rely on rote memorization rather than developing a meaningful understanding of anatomical relationships, which in turn contributes to anxiety and superficial learning strategies (4).

Traditional approaches to anatomy teaching, particularly didactic lectures and cadaveric dissection, present notable pedagogical limitations. Although cadaver-based instruction remains highly valued for its realism, it is increasingly constrained by resource demands, donor availability and curriculum time pressures (1). When used in isolation, large-group lectures and other passive teaching formats tend to promote surface learning, characterized by short-term memorization and poor transfer of knowledge to clinical contexts. Reduced opportunities for hands-on and practical learning may also hinder the development of spatial reasoning skills, further exacerbating students’ difficulties in conceptualizing anatomical structures (4). These challenges have prompted growing calls for a shift toward more student-centered and active learning approaches in anatomy education.

Active learning engages students cognitively through problem-solving, discussions, and frequent testing rather than passive reception of information. These methods consistently outperform traditional passive instruction in health sciences education (5). Retrieval practice via regular low-stakes testing enhances long-term retention of anatomical knowledge by exploiting the testing effect, in which active recall strengthens memory more effectively than repeated study (6). Image-based activities, such as labeling anatomical diagrams and radiological images, capitalize on the visual–spatial nature of anatomy and promote accurate mental models (1). Collectively, these strategies foster deeper cognitive processing, yielding more durable learning outcomes and greater student motivation (4, 5).

Digital technologies have expanded active and self-directed learning in anatomy. Three-dimensional software, virtual dissection tables, and augmented/virtual reality applications, when integrated with traditional methods, provide dynamic visualizations that enhance spatial understanding and enable self-paced engagement (1). These tools support repeated practice, personalized revision, and autonomous learning beyond scheduled sessions, effectively complementing face-to-face instruction (3). Blended approaches combining physical and digital resources appear particularly effective (1).

Assessment design strongly influences learning behaviors in anatomy. Assessments emphasizing factual recall promote surface learning, while those focused on applied understanding and spatial reasoning encourage deeper approaches. Constructive alignment between objectives, teaching activities, and assessment has been shown to improve motivation, engagement, and depth of learning (7). Aligning assessment with active learning tasks thus serves both evaluative and pedagogical purposes.

Finally, motivational factors are central to successful anatomy learning. Educational strategies that promote learner autonomy and provide meaningful feedback to support competence have been associated with greater engagement, persistence and self-directed learning, whereas overly controlling environments may undermine motivation and contribute to disengagement (8). Incorporating autonomy-supportive and competence-enhancing elements into anatomy education may therefore play a key role in improving students’ learning experiences and outcomes.

Accordingly, the present study aimed to evaluate first-year nursing students’ perceptions of the educational value, acceptability, and satisfaction associated with an image-based anatomy self-assessment activity designed to reinforce anatomical identification across the full syllabus under real educational conditions.

## 2 Materials and methods

### 2.1 Study design

This study adopted a cross-sectional, descriptive design embedded within routine course delivery to evaluate students’ perceptions of an image-based anatomy learning activity. The intervention was implemented through an online platform and structured as an individual, asynchronous learning task integrated into the module’s continuous assessment strategy.

**Figure 1:**
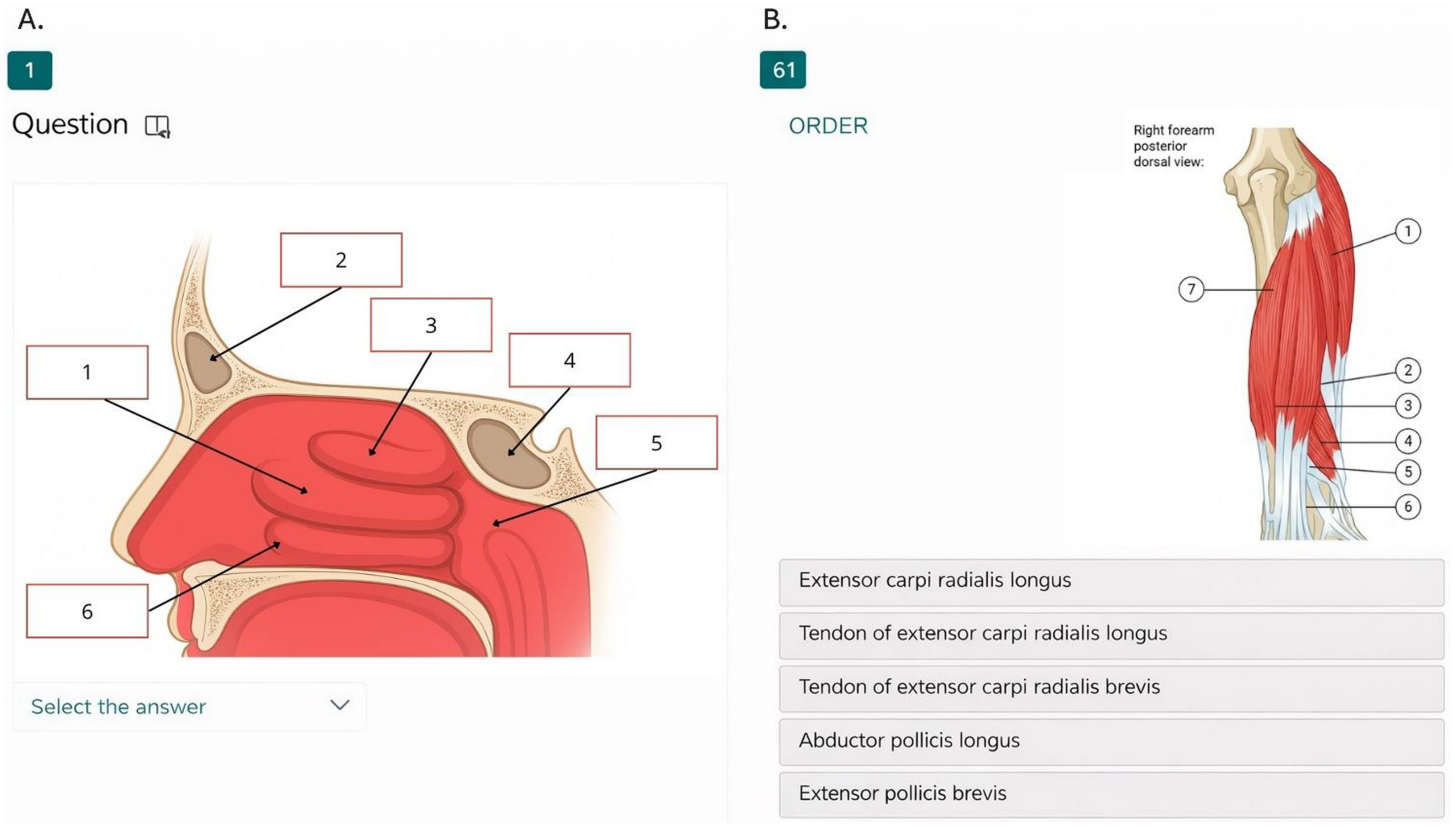
Examples of image-based tasks used in the anatomy learning activity. **(A)** Anatomical identification task requiring students to recognize labeled structures in a sagittal view of the nasal cavity. **(B)** Ordering task requiring students to correctly sequence muscles and tendons of the posterior compartment of the forearm based on an anatomical image.

The learning activity consisted of a total of 266 image-based questions, distributed across the full Human Anatomy syllabus and combining anatomical identification and ordering tasks (Figure 1; see Supplementary material 1). The pedagogical format emphasized repeated engagement with anatomical images and encouraged students to consult multiple learning resources, including course materials and anatomical atlases.

The asynchronous format supported self-paced, flexible, and self-directed learning. Embedded feedback allowed students to monitor progress and reflect on their performance. Longitudinal distribution of the task across the course reinforced anatomical knowledge and aligned with module objectives and assessment requirements.

### 2.2 Educational framework of the intervention

The image-based anatomy identification and sequencing tasks were informed by complementary pedagogical theories addressing the cognitive and motivational demands of anatomy learning. Constructivist learning theory provides the overarching framework, conceptualizing learning as an active process in which students construct knowledge through engagement with content. Active interaction with anatomical images, through identification and sequencing, promotes learner-centered inquiry and multimodal processing, supporting the development of spatial understanding and durable knowledge structures (9). This approach aligns with evidence indicating that active engagement enhances retention in anatomy education (10) and supports spiral learning by enabling progressive knowledge construction over time.

Cognitive Load Theory (CLT) guided the design of the visual materials to optimize information processing. Given the inherent complexity of anatomy, tasks were structured to focus on limited sets of structures, thereby managing intrinsic cognitive load. Extraneous load was minimized through integrated text–image formats and clear visual cues, reducing split-attention and redundancy effects in line with multimedia learning principles (11). The asynchronous format further allows learners to regulate pace according to individual needs, a strategy recommended for complex visual–spatial learning (12). Together, these principles aim to maximize germane cognitive load and facilitate efficient schema construction.

The pedagogical design also draws on experiential and situated learning theories, emphasizing learning through meaningful activity. Identifying anatomical structures in images constitutes a form of active experimentation that engages cognitive processes relevant to clinical practice, such as interpreting diagnostic images or recognizing anatomical landmarks. Experiential learning models, including Kolb’s cycle, support the integration of practice, feedback and reflection, and have been associated with improved clinical skill performance when applied to anatomy education (13). Embedding these activities longitudinally promotes contextualized learning and helps bridge the theory–practice gap commonly reported in anatomy training (10).

Importantly, the framework aligns with competency-based education principles underpinning curricula in the European Higher Education Area. The Bologna Process emphasizes learning outcomes and competencies over content accumulation (14). In European nursing education, this approach prioritizes the application of anatomical knowledge in patient care rather than factual recall alone (15). Accordingly, anatomy is positioned as a core professional competency essential for safe nursing practice, consistent with contemporary educational reform (16).

Additional principles address inclusivity, accessibility and motivation. The asynchronous online format promotes flexibility and equitable participation, accommodating diverse learning needs and external responsibilities. Digital resources were designed following Universal Design for Learning (UDL) principles, providing multiple means of representation and engagement to support diverse learners and enhance accessibility (17).

The framework incorporates motivational principles from educational psychology to sustain engagement in a cognitively demanding subject. Immediate, low-stakes feedback supports perceived competence, while learner autonomy is fostered through flexible pacing and individual engagement. Interactive, clinically relevant image-based tasks enhance instrumental motivation by reinforcing the relevance of anatomy for professional practice (18). Regular formative assessment distributed over time further supports sustained engagement and long-term retention through spaced retrieval practice (19).

### 2.3 Participants

Participants were first-year undergraduate students enrolled in the Human Anatomy module of the Bachelor’s Degree in Nursing at a European university in Valencia during the academic year 2025–2026. All students registered in the module were invited to take part in the image-based anatomy learning activity, which formed part of the module’s continuous assessment strategy. The same image-based activity was independently implemented at two campuses (Valencia and Alicante) of the same institution. Campus-level comparisons were conducted to evaluate the consistency of student perceptions across independent implementations rather than to test between-group hypotheses.

Students were included in the study if they had completed the learning activity and subsequently submitted the post-activity evaluation questionnaire.

### 2.4 Variables

The study included sociodemographic variables (sex, age, nationality, campus, access pathway, and academic status), as well as performance and satisfaction outcomes. Age was analyzed both as a continuous measure and as grouped categories (<21, 21–25, >25). Campus was classified as Valencia or Alicante. Nationality was categorized as Spanish versus foreign. Access pathway was grouped into new entry, vocational training, and prior university, and academic status distinguished first enrollment from students with prior credits.

Performance outcomes consisted of the activity score (0–10) and the total number of correct answers on the image-based anatomy activity. Satisfaction was measured using the Net Promoter Score (NPS, 0–10), and NPS categories were defined as detractors (0–6), passives (7–8), and promoters (9–10).

### 2.5 Data analysis

Data were cleaned to include only students who completed both the activity and the post-activity questionnaire. Descriptive statistics (means, standard deviations, medians, IQRs, and percentages) were used to summarize all variables. Normality was assessed using the Shapiro–Wilk test; all continuous variables violated normality (all *p* < 0.001; Supplementary Table S3), so non-parametric methods were adopted throughout (*α* = 0.05). Mann–Whitney U tests were applied for two-group comparisons, and Kruskal–Wallis tests for variables with more than two categories, with rank-biserial *r* and epsilon-squared (ε^2^) as effect-size measures, respectively. Correlations were assessed using Spearman’s rho.

Student satisfaction was measured on a 0–10 scale and classified as Detractors (0–6), Passives (7–8), or Promoters (9–10). The NPS index was computed as %Promoters minus %Detractors (range −100 to +100) (20), with a 95% bootstrap confidence interval (10,000 replications).

To identify student profiles, k-means clustering was performed on two Z-standardized variables (activity score and NPS). The optimal number of clusters was selected by comparing elbow, silhouette, and gap-statistic criteria (Supplementary Figure 3) and validated against Ward’s hierarchical clustering (Adjusted Rand Index). Clusters were subsequently characterized by demographic and academic attributes as post-hoc descriptors (Supplementary Table S6).

Three complementary regression models, linear, ordinal logistic (cumulative logit), and multinomial logistic, were fitted to assess the association between NPS and student characteristics (Supplementary Tables S7, S8).

Open-ended comments were analyzed through a two-stage content-based approach: rule-based sentiment classification using Spanish-language lexicons with negation handling, followed by thematic coding with predefined dictionaries. Coherence between sentiment and NPS category was assessed, and discrepant cases were reviewed individually (Supplementary Table S5). This exploratory approach does not constitute a formal qualitative methodology.

All statistical analyses were conducted using R v4.5.2 (R Foundation for Statistical Computing) using the packages dplyr, ggplot2, cluster, factoextra, boot, moments, MASS, and nnet.

## 3 Results

### 3.1 Participants and data quality

Of 171 enrolled students, 159 (93.0%) completed the activity and provided a valid NPS response. The 12 excluded cases had no activity data, confirming non-participation rather than selective non-response (Supplementary Table S1). All continuous variables violated normality (Shapiro–Wilk *p* < 0.001), establishing non-parametric methods as default (Supplementary Tables S2–S5). A comparison of respondents and non-respondents revealed no meaningful differences in age, sex distribution, or access pathway (Supplementary Tables S1, S4).

### 3.2 Sample characteristics

The sample was predominantly female (81.8%), young (median 21 years), and Spanish (95.6%), distributed across Valencia (*n* = 119) and Alicante (*n* = 40). The main between-campus difference was access pathway: new university entrants predominated in Alicante (62.5%) while vocational training was the dominant route in Valencia (58.0%). Full demographics are presented in Table 1.

**Table 1:** Sample characteristics by campus (*N* = 159).

Variable	Category	Valencia	Alicante	Total
*N*		119	40	159
Sex	Female	99 (83.2%)	31 (77.5%)	130 (81.8%)
	Male	20 (16.8%)	9 (22.5%)	29 (18.2%)
Age (years)	Mean ± SD	23.02 ± 5.21	21.7 ± 4.26	22.69 ± 5.01
	Median (IQR)	22 (19–25)	21 (18–23)	21 (18–25)
	Range	18–39	18–37	18–39
Age group	Young (<21)	46 (38.7%)	19 (47.5%)	65 (40.9%)
	Middle (21–25)	49 (41.2%)	15 (37.5%)	64 (40.3%)
	Older (>25)	24 (20.2%)	6 (15%)	30 (18.9%)
Nationality	Spanish	112 (94.1%)	40 (100%)	152 (95.6%)
	Foreign	7 (5.9%)	0 (0%)	7 (4.4%)
Access pathway	New entry	38 (31.9%)	25 (62.5%)	63 (39.6%)
	Vocational training	69 (58%)	11 (27.5%)	80 (50.3%)
	Prior university	12 (10.1%)	4 (10%)	16 (10.1%)
Academic status	First enrollment	112 (94.1%)	39 (97.5%)	151 (95%)
	With prior credits	7 (5.9%)	1 (2.5%)	8 (5%)
Province	Valencia	96 (80.7%)	2 (5%)	98 (61.6%)
	Alicante	3 (2.5%)	37 (92.5%)	40 (25.2%)
	Other	20 (16.8%)	1 (2.5%)	21 (13.2%)
Activity score	Mean ± SD	8.36 ± 1.16	8.82 ± 0.75	8.48 ± 1.09
	Median (IQR)	8.72 (7.97–9.21)	9.04 (8.42–9.47)	8.8 (8.2–9.21)
	Range	4.14–9.66	6.73–9.77	4.14–9.77
Correct answers	Mean ± SD	222.48 ± 30.87	234.68 ± 20.08	225.55 ± 28.97
	Median (IQR)	232 (212–245)	240.5 (224–252)	234 (218–245)
	Range	110–257	179–260	110–260
NPS	Mean ± SD	8 ± 2.1	7.9 ± 2.02	7.97 ± 2.07
	Median (IQR)	9 (7–10)	8 (7–10)	8 (7–10)
	Range	2–10	2–10	2–10

Activity scores were high overall (median 8.8/10, IQR 8.2–9.2), with a 98.1% pass rate and a pronounced ceiling effect, with scores concentrated toward the upper end of the scale.

### 3.3 Satisfaction: net promoter score

**Figure 2:**
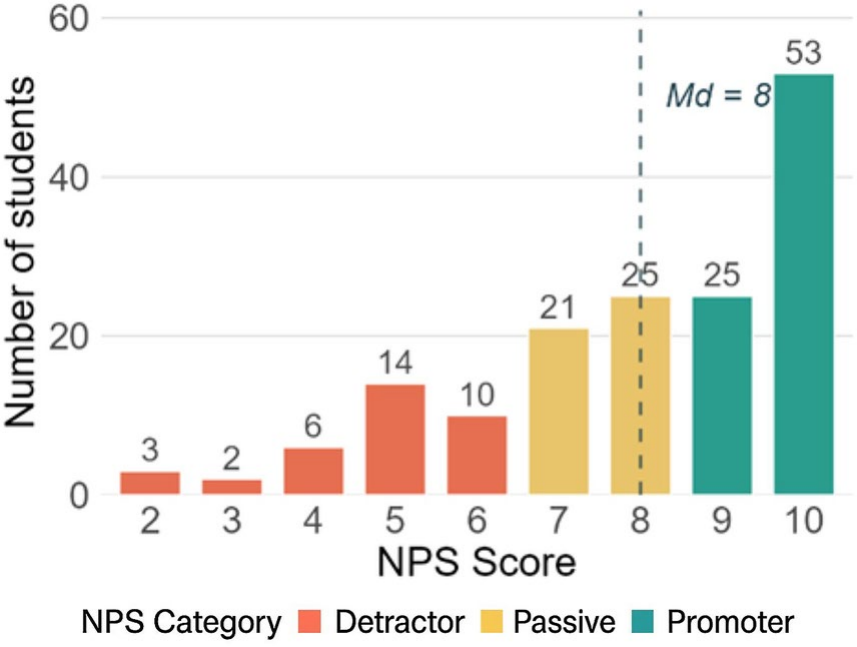
Distribution of individual NPS scores (*N* = 159). Each bar represents the number of students assigning that score. Bars are colored by NPS category: Detractors (0–6), Passives (7–8), and Promoters (9–10). The dashed vertical line indicates the median score (Md = 8). Qualitative feedback.

The overall NPS index was +27 (95% bootstrap CI: 14.5–39.6), with 49.1% Promoters, 28.9% Passives, and 22.0% Detractors (Figure 2; Table 2). Valencia showed a slightly higher NPS (+28.6) than Alicante (+22.5), driven by a larger Promoter share (52.1% vs. 40.0%).

**Table 2:** NPS distribution by campus and sex.

Variable	Valencia	Alicante	Female	Male	Total
Median (IQR)	9 (7–10)	8 (7–10)	9 (7–10)	8 (7–10)	8 (7–10)
Promoters	52.1%	40.0%	51.5%	37.9%	49.1%
Passives	24.4%	42.5%	24.6%	48.3%	28.9%
Detractors	23.5%	17.5%	23.8%	13.8%	22%
NPS Index	+28.6	+22.5	+27.7	+24.1	+27.1

95% bootstrap CI for overall NPS: 14.5–39.6 (10,000 replications).

**Figure 3:**
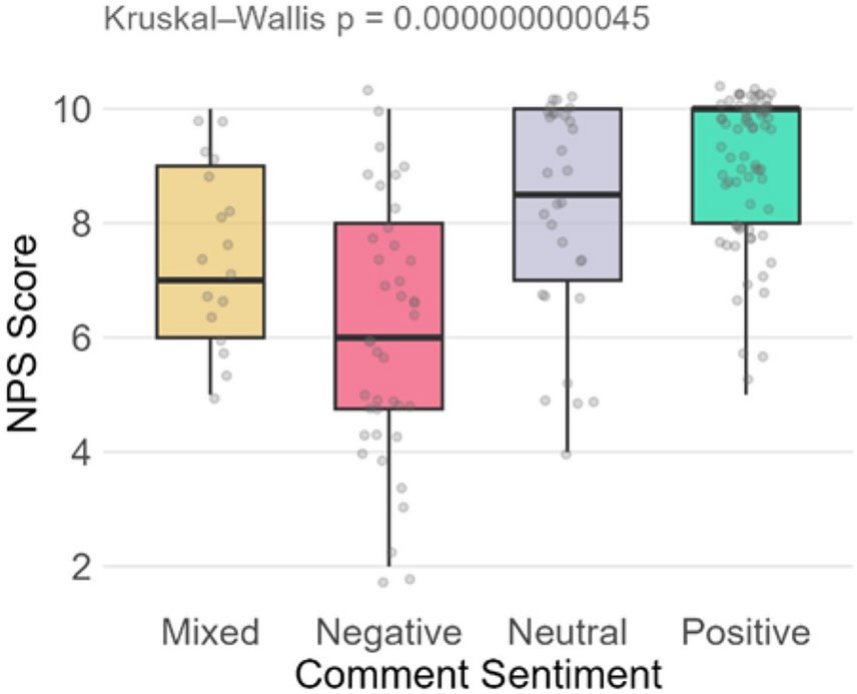
Distribution of NPS scores by comment sentiment (*N* = 159). Comments were classified into four categories (Positive, Mixed, Negative, Neutral) using a rule-based Spanish-language lexicon approach with negation handling. Boxes represent median and interquartile range; whiskers extend to 1.5 × IQR; individual observations are overlaid as jittered points. A Kruskal–Wallis test was performed to assess whether NPS scores differed significantly across sentiment categories.

Of the 159 respondents, 100% provided a written comment. Character number: 4 min, median 109, max 576. Open-ended comments were processed through a two-stage NLP pipeline (sentiment classification and thematic coding). Positive sentiment predominated (40.3%), and mean NPS scores followed a monotonic gradient across sentiment groups (positive: 9.17, neutral: 8.20, mixed: 7.29, negative: 6.15; Kruskal-Wallis *p* < 0.001). The overall coherence between NPS ratings and comment sentiment was 93.7%, validating the internal consistency of the satisfaction measure (Figure 3).

Perceived utility was the most frequently mentioned theme (59.1%), followed by four critical areas: image quality (26.4%), errors in answers (22.6%, mean NPS 6.32), formatting issues (22.0%), and excessive length (19.5%, mean NPS 6.74). These critical themes identify specific, correctable aspects of the tool that drive dissatisfaction (Table 3).

**Table 3:** Principal themes in student comments (*N* = 159).

Theme	*n*	%	Mean NPS	Md NPS
Perceived utility	94	59.1	8.54	9
Image quality concerns	42	26.4	7.67	8
Errors/ambiguity	36	22.6	6.32	7
Formatting issues	35	22.0	—	—
Excessive length	31	19.5	6.74	7

Multiple themes per comment permitted. Only the five most frequent themes shown.

### 3.4 Student profiles

**Figure 4:**
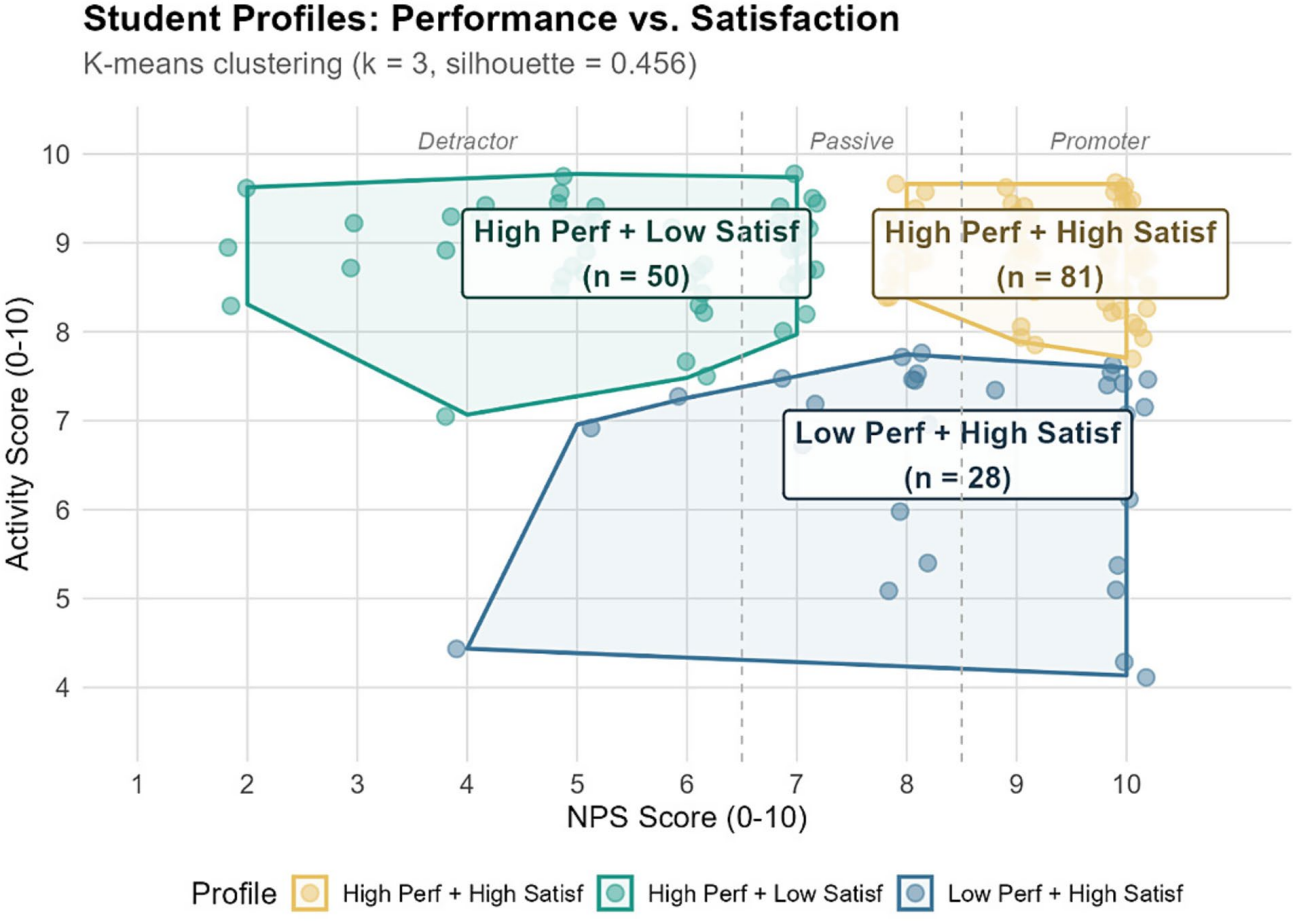
Student profiles by NPS and activity score (*N* = 159). Shaded areas: convex hulls. Dashed lines: NPS category thresholds.

K-means clustering on activity score and NPS (both Z-standardized) identified three profiles (Figure 4; Table 4). The Engaged Learner profile (51%) combined high scores (*M* = 8.87) with high satisfaction (*M* = 9.31). The Critical Achiever profile (31%) achieved identical scores (*M* = 8.88) but reported substantially lower satisfaction (*M* = 5.54), aligning with the critical themes in section 3.4. The Effort Appreciator profile (18%) scored lower (*M* = 6.62) but still valued the tool (*M* = 8.46), predominantly comprising vocational training students (57.1%).

**Table 4:** Student profile characterization (*N* = 159).

Characteristic	Engaged learner	Critical achiever	Effort appreciator	Total
*n* (%)	81 (51%)	50 (31%)	28 (18%)	159
Activity score, M	8.87	8.88	6.62	8.5
NPS, M	9.31	5.54	8.46	8.0
Alicante, %	—	28	14	25
Vocational Tr., %	—	—	57	44

Supplementary Table S6 reports full demographic characterization per profile.

The absence of a “low score + low satisfaction” profile indicates that dissatisfaction is not driven by poor results but by experiential factors. The horizontal split between Profiles 1 and 2, same performance, different satisfaction, makes the Critical Achiever the primary target for improvement.

### 3.5 Factors associated with satisfaction

Bivariate tests (Mann–Whitney U, Kruskal-Wallis, Spearman) and three multivariate models (linear, ordinal, multinomial regression) converged on the same finding: no demographic or academic variable significantly predicted NPS. All bivariate comparisons yielded *p* > 0.15 with negligible effect sizes, and NPS was uncorrelated with activity score (*ρ* = −0.094, *p* = 0.238). The three regression models explained near-zero variance (R^2^ range: −0.01 to 0.056). The only significant predictor was sex in the multinomial model (female OR = 2.81 for Promoter vs. Passive, *p* = 0.030), to be interpreted cautiously given sample imbalance (130 vs. 29). This homogeneity across subgroups indicates that satisfaction is driven by experiential factors rather than student characteristics, consistent with the qualitative findings above (Table 5; Supplementary Tables S7, S8).

**Table 5:** Bivariate associations between NPS and student characteristics (*N* = 159).

Comparison	Test	Statistic	p_value	Effect_size	Interpretation
NPS × Sex	Mann–Whitney U	U = 1894	0.969	*r* = 0.005	Negligible
NPS × Campus	Mann–Whitney U	U = 2,280	0.686	*r* = 0.042	Negligible
NPS × Nationality	Mann–Whitney U	U = 585	0.651	*r* = 0.1	Negligible
NPS × Prior credits	Mann–Whitney U	U = 763.5	0.199	*r* = 0.264	Small
NPS × Age group	Kruskal-Wallis	H(2) = 1.64	0.44	eps2 = 0.0104	Small
NPS × Access pathway	Kruskal-Wallis	H(2) = 3.776	0.151	eps2 = 0.0239	Small
NPS × Activity score	Spearman	rho = −0.094	0.238	rho = −0.094	Negligible
NPS × Age	Spearman	rho = 0.09	0.26	rho = 0.09	Negligible
NPS × Credits passed	Spearman	rho = −0.101	0.206	rho = −0.101	Weak
Activity Score × Age	Spearman	rho = −0.052	0.517	rho = −0.052	Negligible
Score × Credits passed	Spearman	rho = −0.059	0.459	rho = −0.059	Negligible
Age × Credits passed	Spearman	rho = 0.182	0.0214	rho = 0.182	Weak

## 4 Discussion

The results of the present study indicate that structured, image-based self-assessment is a highly accepted and educationally valuable strategy for nursing students. This high level of satisfaction mirrors findings in other anatomical education contexts; for instance, the “Virtual Quiz” platform was consulted by 89.5% of registered students with very positive qualitative satisfaction levels (21). Similarly, research on medical students using online self-assessment tools during the COVID-19 pandemic reported that 85% of participants found the method useful, interesting, and easy (22). The use of NPS in this study as a metric of value is supported by literature suggesting that NPS in medical education is a valid indicator of a learner’s intention to act or change behavior based on the educational experience (20).

Regarding sample characteristics and demographics, our finding that gender did not significantly influence satisfaction or academic performance is consistent with Bradley et al. (23) who found that gender and age did not influence satisfaction levels with active learning techniques in online graduate anatomy. This result may also be interpreted in light of broader evidence showing that anatomy study preferences can vary according to academic year and geographical context, while gender appears to play a more limited role (24). In our study, neither sex nor campus significantly influenced satisfaction or performance, which may reflect the relative homogeneity of the sample and the standardized implementation of the activity across settings. Also, literature suggests that older students may prefer traditional lectures over active digital strategies (23), the relative homogeneity of our young, predominantly female sample aligns with the typical demographic profile of nursing education where digital literacy is generally high (25).

The activity scores in this study demonstrated a pronounced ceiling effect, with a 98.1% pass rate and a median score of 8.8/10. This pattern is typical for formative assessment tools designed to reinforce identification through repetition. Ribeiro et al. similarly demonstrated that students who utilized virtual quiz tools achieved significantly higher classifications in practical examinations (identification of structures) compared to those who did not, although no differences were found in theoretical exams (21). This suggests that such tools are specifically effective for topographic and functional identification rather than broad theoretical mastery (21, 25). This interpretation is further supported by evidence indicating that individual study and regular repetition are among the most common approaches to anatomy learning (24). The positive reception of our activity may therefore be explained not only by its digital format, but also by its alignment with these established study habits, particularly repeated review and self-paced engagement with visual content.

In the qualitative feedback, the most frequent theme was perceived utility (59.1%), with students valuing the activity for reinforcing anatomical knowledge. This finding is supported by Pettersson et al. (26) where students reported that digital resources provided essential tools for repetition and memorization, simplifying the learning process and complementing traditional textbooks. Our students highlighted the role of these tools in reviewing content, which aligns with the observation that most students use digital anatomy resources specifically to prepare for examinations or when encountering difficult concepts.

However, students in our study also reported barriers such as technical limitations, image clarity issues, and an excessive workload. These criticisms are common in technology-enhanced learning (TEL) research. Previous studies have noted that “heavy” digital resources can cause computer lag, leading to feelings of ineffectiveness (26). Furthermore, the reported concern regarding workload is corroborated by the TraceX randomized controlled trial, where intervention students spent significantly more time in the learning module than the control group, suggesting that integrated digital courses often impose a higher time demand on learners (25).

Finally, the student profiles identified through cluster analysis, specifically the Engaged Learner and Critical Achiever, illustrate that satisfaction is driven by experiential factors rather than purely academic results. This is consistent with findings that student satisfaction in online anatomy is influenced more by course design and teaching quality than by preexisting knowledge or grades (27). The presence of “Critical Achievers” who perform well but report low satisfaction highlights the need for educators to provide an introduction to these resources and ensure high-quality, unambiguous visual materials to prevent frustration (26). Strengthening visually supported and interactive feedback mechanisms may further optimize the efficacy of such self-assessment tools in nursing education (28).

## 5 Limitations

This study has some limitations that should be considered when interpreting the findings. First, its cross-sectional design, together with the absence of a control group and pre-post comparison, prevents establishing causal relationships between the intervention and improvements in anatomical knowledge; however, this design allowed the evaluation of the intervention under real educational conditions and provides relevant evidence regarding its feasibility and acceptability in routine practice. Although performance scores were high, the lack of baseline or comparative data limits the ability to determine whether these results reflect true learning gains or are influenced by the formative nature of the activity, which was intentionally designed to support reinforcement rather than summative assessment. Second, the study relies primarily on self-reported measures of satisfaction and perceived educational value, including the NPS; while this does not capture the full complexity of learning processes, it offers a simple and interpretable indicator of student experience that is increasingly explored in educational contexts. Future studies could strengthen this aspect by incorporating complementary validated educational instruments, such as the Course Experience Questionnaire (CEQ) or the Students’ Evaluation of Educational Quality (SEEQ), to provide a more comprehensive and standardized assessment of the learning experience. Third, the qualitative component was based on a single open-ended question and descriptive analysis, which may have constrained the depth of student perspectives, although it still provided consistent and actionable insights into key aspects of the learning experience, such as perceived utility and usability issues. In addition, the study did not include objective measures of long-term knowledge retention or transfer to clinical performance, which are important outcomes in anatomy education, but it does provide a valuable initial understanding of how students engage with and perceive structured image-based self-assessment activities. Finally, the possibility of selection bias cannot be excluded, as students who completed both the activity and the evaluation may have been more engaged; nevertheless, the high participation rate supports the representativeness of the sample within this educational context, although the single-institution setting may limit generalisability. Future research should build on these findings through longitudinal, multi-institutional designs incorporating objective learning outcomes and more robust mixed-methods approaches.

## 6 Conclusion

This study shows that a structured, image-based self-assessment activity is well accepted by first-year nursing students and is perceived as educationally useful. Students reported high satisfaction levels, reflected in a positive Net Promoter Score and consistently strong activity scores, indicating that the tool effectively supports the reinforcement of anatomical identification skills. Qualitative feedback further emphasized its utility for reviewing and consolidating course content, while also highlighting areas in need of improvement, such as image clarity, technical performance, and overall workload. Importantly, neither satisfaction nor performance differed across demographic or academic subgroups, suggesting that the activity provides a comparable learning experience for diverse student profiles. These findings support the incorporation of image-based self-assessment activities as a complementary component within undergraduate anatomy instruction in nursing education, offering students new ways to interact with and understand the human body.

## Data Availability

The original contributions presented in the study are included in the article/Supplementary material, further inquiries can be directed to the corresponding author.
