# Supplementary material for: Nursing students’ perceptions of image-based anatomical self-assessment in undergraduate education
Source: Front Med (Lausanne). 2026 May 25;13:1826706. doi: 10.3389/fmed.2026.1826706 (PMC13244495; doi:10.3389/fmed.2026.1826706)
Supplement: Supplementary file 1 [file Data_Sheet_1.docx]

Supplementary Material 1

# Supplementary Data

## Supplementary Tables

**Supplementary Table S1.** Non-respondent characteristics (the 12 excluded cases)

| **id** | **correct_answers** | **activity_score** | **nps** | **comments** | **academic_year** | **nationality_original** | **access_pathway_original** | **age** | **sex** | **birth_country** | **campus_code** | **credits_passed** | **city_original** |
| --- | --- | --- | --- | --- | --- | --- | --- | --- | --- | --- | --- | --- | --- |
| 4 |  |  |  |  | 1 | SPANISH | University Entrance Examination | 19 | Female | SPAIN | VAL | 0 | València |
| 5 |  |  |  |  | 1 | SPANISH | Transfer of Academic Record (different studies) | 20 | Female | SPAIN | VAL | 96 | Dénia |
| 10 |  |  |  |  | 2 | SPANISH | Advanced Vocational Training Programs | 25 | Female | SPAIN | VAL | 36 | Gandia |
| 31 |  |  |  |  | 1 | SPANISH | University Entrance Examination | 22 | Female | SPAIN | VAL | 48 | Bétera |
| 61 |  |  |  |  | 1 | SPANISH | Advanced Vocational Training Programs | 20 | Male | SPAIN | VAL | 0 | Real |
| 62 |  |  |  |  | 1 | SPANISH | Advanced Vocational Training Programs | 38 | Female | SPAIN | VAL | 0 | Valencia |
| 71 |  |  |  |  | 1 | SPANISH | Advanced Vocational Training Programs | 20 | Female | SPAIN | VAL | 0 | La Pobla de Vallbona |
| 81 |  |  |  |  | 2 | SPANISH | Transfer of Academic Record | 26 | Female | SPAIN | VAL | 0 | Riba Roja |
| 82 |  |  |  |  | 1 | SPANISH | University Entrance Examination | 18 | Female | SPAIN | VAL | 0 | València |
| 102 |  |  |  |  | 1 | SPANISH | Advanced Vocational Training Programs | 19 | Female | SPAIN | VAL | 0 | Godella |
| 112 |  |  |  |  | 1 | SPANISH | University Entrance Examination | 21 | Female | SPAIN | VAL | 0 | Oliva |
| 132 |  |  |  |  | 2 | SPANISH | University Entrance Examination | 22 | Male | SPAIN | ALI | 36 | Motril |

**Supplementary Table S2.** Individual outlier case details

| **id** | **activity_score** | **age** | **nps** | **campus** | **outlier_score_iqr** | **outlier_age_iqr** | **outlier_nps_iqr** |
| --- | --- | --- | --- | --- | --- | --- | --- |
| 7 | 8,95 | 19 | 2 | Valencia | 1 | 0 | 1 |
| 8 | 6,02 | 21 | 8 | Valencia | 1 | 0 | 0 |
| 12 | 8,72 | 38 | 6 | Valencia | 1 | 0 | 0 |
| 16 | 4,44 | 25 | 4 | Valencia | 1 | 0 | 0 |
| 18 | 8,8 | 38 | 10 | Valencia | 0 | 1 | 0 |
| 20 | 5,38 | 20 | 8 | Valencia | 1 | 0 | 0 |
| 22 | 8,23 | 37 | 7 | Valencia | 1 | 0 | 0 |
| 24 | 8,72 | 36 | 10 | Valencia | 1 | 0 | 0 |
| 30 | 6,17 | 18 | 10 | Valencia | 1 | 0 | 0 |
| 34 | 8,31 | 28 | 2 | Valencia | 1 | 0 | 1 |
| 39 | 5,08 | 21 | 8 | Valencia | 1 | 0 | 0 |
| 46 | 8,83 | 38 | 10 | Valencia | 1 | 0 | 0 |
| 48 | 5,11 | 25 | 10 | Valencia | 1 | 0 | 0 |
| 71 | 7,48 | 39 | 10 | Valencia | 0 | 1 | 0 |
| 85 | 4,29 | 19 | 10 | Valencia | 1 | 0 | 0 |
| 116 | 5,34 | 21 | 10 | Valencia | 1 | 0 | 0 |
| 118 | 4,14 | 33 | 10 | Valencia | 1 | 0 | 0 |
| 134 | 9,62 | 20 | 2 | Alicante | 0 | 0 | 1 |
| 140 | 8,8 | 37 | 8 | Alicante | 0 | 1 | 0 |

**Supplementary Table S3.** Normality summary: W, p, skewness, kurtosis, recommended test

| **variable** | **n** | **mean** | **median** | **sd** | **skewness** | **kurtosis** | **shapiro_w** | **shapiro_p** | **shapiro_p_fmt** | **is_normal** | **recommended_test** |
| --- | --- | --- | --- | --- | --- | --- | --- | --- | --- | --- | --- |
| activity_score | 159 | 8,479 | 8,797 | 1,089 | -1,89 | 6,975 | 0,813 | 5,5932E-13 | 5,59E-12 | FALSE | Mann-Whitney / Kruskal-Wallis |
| nps | 159 | 7,975 | 8 | 2,071 | -0,9 | 3,021 | 0,865 | 9,1098E-11 | 9,11E-11 | FALSE | Mann-Whitney / Kruskal-Wallis |
| age | 159 | 22,69 | 21 | 5,01 | 1,409 | 4,656 | 0,837 | 4,9255E-12 | 4,93E-12 | FALSE | Spearman |

**Supplementary Table S4.** Non-response comparison (respondents vs. non-respondents)

| **variable** | **respondents** | **non_respondents** |
| --- | --- | --- |
| n | 159 | 12 |
| age_mean | 22,69 | 22,42 |
| age_sd | 5,01 | 5,45 |
| score_mean | 8,48 |  |
| score_sd | 1,09 |  |
| pct_female | 81,8 | 75 |

**Supplementary Table S5.** Incoherent case details (9 cases with ID, NPS, sentiment, comment)

| **interference_type** | **id** | **nps** | **nps_category** | **sentiment** | **n_pos_words** | **n_neg_words** | **comments_en** |
| --- | --- | --- | --- | --- | --- | --- | --- |
| T1: Promoter + Negative | 41 | 10 | Promoter | Negative | 0 | 1 | There is an error in the image of the bladder; it still shows the internal urethral sphincter without exiting, so in two of the images I placed it externally. |
| T1: Promoter + Negative | 54 | 9 | Promoter | Mixed_Negative | 2 | 1 | In my opinion it has been too extensive and some of the images were not well numbered, but removing that, I think it is a useful tool. |
| T1: Promoter + Negative | 59 | 9 | Promoter | Negative | 0 | 2 | I think it is very useful for studying but very costly and too extensive for an assignment; I would reduce some images. Also, the numbers are poorly structured, in some images it goes from 8 to 15; please review. Best regards. |
| T1: Promoter + Negative | 64 | 9 | Promoter | Negative | 0 | 1 | too much |
| T1: Promoter + Negative | 82 | 10 | Promoter | Negative | 0 | 2 | The completion of the sheets in my opinion has been quite demanding, although they do serve to study and review. Things to consider and that should be changed are that when you make them they are not saved, so if you close it everything is erased. In addition, there are images that were not entirely clear. |
| T1: Promoter + Negative | 113 | 9 | Promoter | Negative | 1 | 0 | It is practical to review and have fun studying anatomy; although at the same time the online method with the answer tabs is tedious, since errors from the previous tasks are not hidden and the same answers reappear. |
| T1: Promoter + Negative | 133 | 9 | Promoter | Negative | 0 | 1 | A little confusing with the numbers and the order of the first part. |
| T2: Detractor + Positive | 147 | 6 | Detractor | Positive | 1 | 0 | Good. |
| T5: NPS>=9 + Net Negative | 95 | 9 | Promoter | Mixed_Balanced | 2 | 3 | The activity itself was very good for review, but the organization of the online sheet with so many errors or problems when doing it, including having to reach the end with everything done and putting the exercise number, searching for it and inserting it, and suddenly the page said everything had been saved. Very frustrating and a waste of time in that aspect. |

**Supplementary Table S6.** Profile characterization (means, SDs, demographics)

| **cluster** | **n** | **profile** | **score_mean** | **score_sd** | **nps_mean** | **nps_sd** | **score_z_mean** | **nps_z_mean** | **age_mean** | **age_sd** | **pct_female** | **pct_alicante** | **pct_vocational** | **pct_new_entry** |
| --- | --- | --- | --- | --- | --- | --- | --- | --- | --- | --- | --- | --- | --- | --- |
| 1 | 50 | High Perf + Low Satisf | 8,88 | 0,57 | 5,54 | 1,47 | 0,37 | -1,18 | 22,7 | 5,2 | 82 | 28 | 42 | 46 |
| 2 | 81 | High Perf + High Satisf | 8,87 | 0,47 | 9,31 | 0,78 | 0,36 | 0,64 | 22,5 | 5 | 85,2 | 27,2 | 53,1 | 38,3 |
| 3 | 28 | Low Perf + High Satisf | 6,62 | 1,15 | 8,46 | 1,67 | -1,17 | 0,24 | 23,1 | 4,9 | 71,4 | 14,3 | 57,1 | 32,1 |

**Supplementary** **Table S7.** Model comparison summary

| **model** | **nps_treatment** | **method** | **n** | **r2_type** | **r2_value** | **aic** | **bic** | **notes** |
| --- | --- | --- | --- | --- | --- | --- | --- | --- |
| A: Linear | Continuous | OLS | 159 | R-squared | 0,0348 | 694,2 | 721,8 | Adj R2=-0.01 |
| B: Ordinal | Ordinal (0-10) | Cumulative logit (polr) | 159 | McFadden R2 | 0,0127 | 613 | 659 | Nagelkerke=0.0473 |
| C: Multinomial | 3-category | Multinomial logit (nnet) | 159 | McFadden R2 | 0,0555 | 344,8 | 393,9 | Ref=Passive |

**Supplementary** **Table S8**. Consolidated coefficients (linear + ordinal)

| **variable** | **lm_beta** | **lm_ci_lower** | **lm_ci_upper** | **lm_p** | **lm_std** | **ord_or** | **ord_ci_lower** | **ord_ci_upper** | **ord_p** |
| --- | --- | --- | --- | --- | --- | --- | --- | --- | --- |
| access_pathway_recodedPrior University | -0,111 | -1,353 | 1,131 | 0,86 | -0,05 | 0,929 | 0,324 | 2,662 | 0,891 |
| access_pathway_recodedVocational Training | 0,408 | -0,436 | 1,252 | 0,341 | 0,197 | 1,531 | 0,74 | 3,169 | 0,251 |
| activity_score | -0,187 | -0,497 | 0,123 | 0,234 | -0,1 | 0,838 | 0,636 | 1,105 | 0,21 |
| age | 0,019 | -0,06 | 0,098 | 0,638 | 0,045 | 1,025 | 0,957 | 1,099 | 0,478 |
| campusAlicante | 0,097 | -0,703 | 0,896 | 0,812 | 0,047 | 1,169 | 0,537 | 2,128 | 0,85 |
| prior_credits_statusWith Prior Credits | -0,914 | -2,452 | 0,624 | 0,202 | -0,44 | 0,417 | 0,118 | 1,467 | 0,173 |
| sexM | -0,129 | -0,981 | 0,723 | 0,765 | -0,06 | 1,102 | 0,546 | 2,223 | 0,787 |

## Supplementary Figures

**
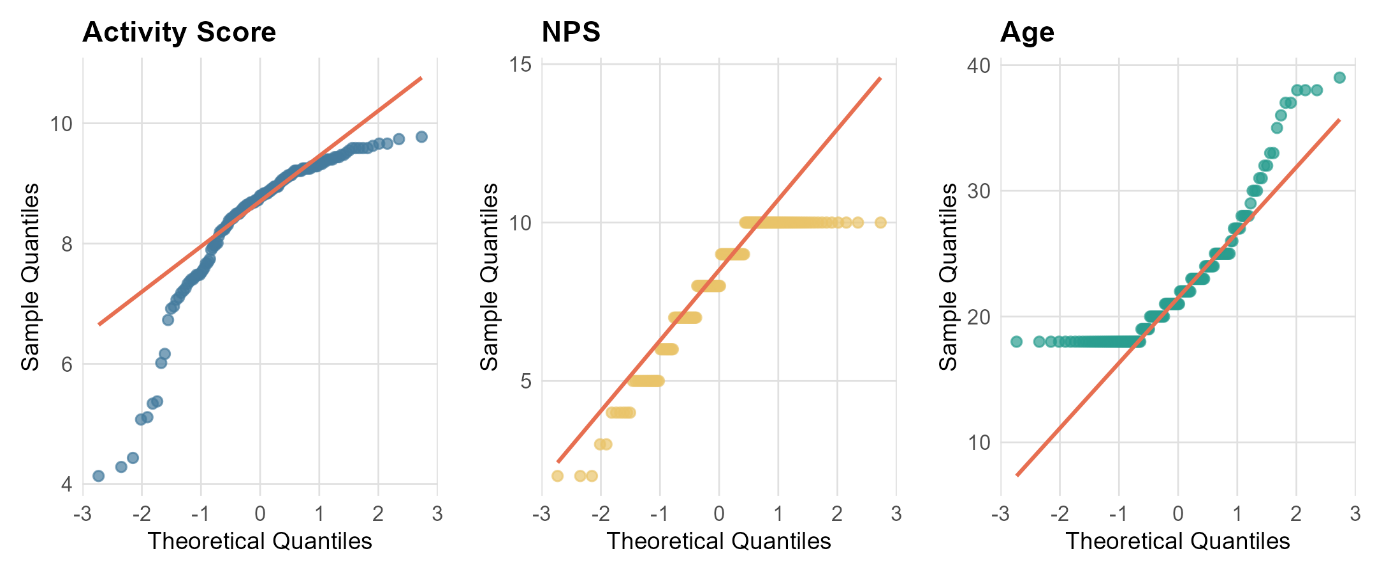
**

**Supplementary Figure 1.** Q-Q plots for the three continuous variables: Q-Q plots for the three continuous variables (N = 159). Deviations from the reference line confirm non-normality across all variables


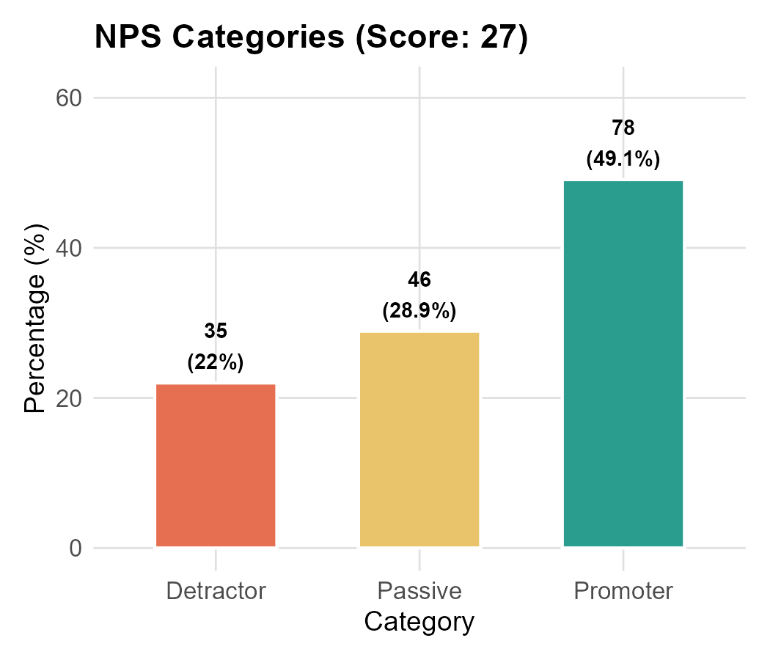


**Supplementary Figure 2.** NPS category bar chart (Detractor/Passive/Promoter counts)


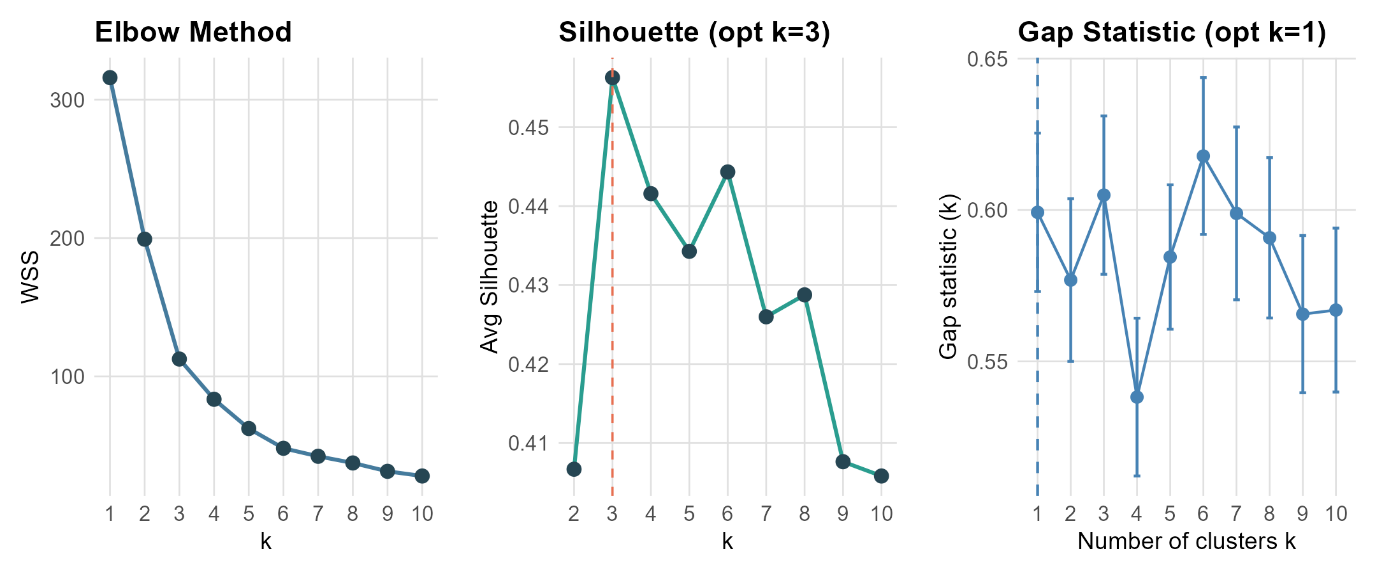


**Supplementary Figure 3.** K-selection methods panel (elbow, silhouette, gap)


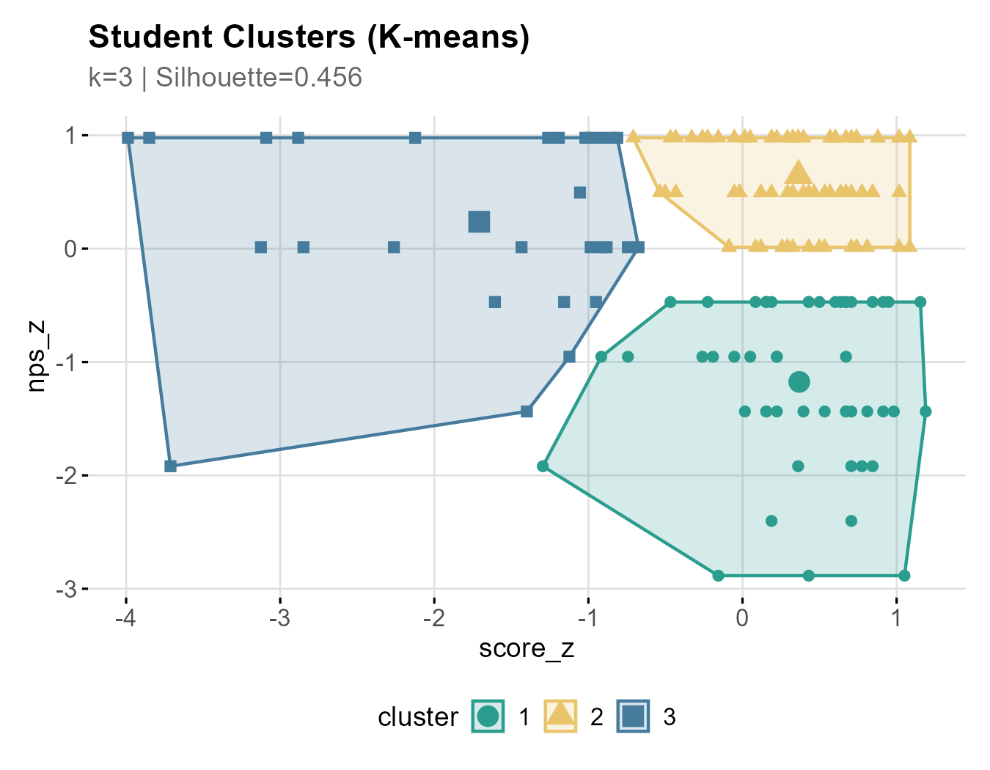


**Supplementary Figure 4.** PCA biplot with cluster membership.

## Content-Based Analysis of Open-Ended Comments: Pipeline, Lexicons, and Validation

### Overview

This document describes the methodology used to analyze the open-ended comments provided by students in the post-activity evaluation questionnaire. A two-stage, rule-based content analysis pipeline was implemented in R to classify each comment by sentiment (valence) and thematic content (topics mentioned). This approach is exploratory and does not constitute a formal qualitative methodology (e.g., thematic analysis or grounded theory). Rather, it provides a structured, reproducible, and transparent method for extracting descriptive patterns from short free-text responses.

All 159 respondents (100%) provided a written comment. Comments ranged from 4 to 576 characters in length (median: 109 characters). All analyses were conducted using R (v4.5.2) with the packages stringr, stringi, and dplyr.

### Text Preprocessing

Before classification, each comment underwent the following normalization steps:

Step 1. Conversion to lowercase.

Step 2. Transliteration of accented characters to ASCII equivalents (e.g., á → a, ñ preserved) using stri_trans_general(text, "Latin-ASCII").

Step 3. Removal of all non-alphanumeric characters except spaces (punctuation, special characters).

Step 4. Whitespace normalization (collapsing multiple spaces into one).

These steps ensure consistent matching regardless of variations in capitalization, accent usage, or punctuation in the original student responses.

### Sentiment Classification

Sentiment was classified using a rule-based approach with two custom lexicons of Spanish-language patterns (regular expressions) designed to capture the evaluative language typically used by university students in course feedback contexts.

#### Positive Lexicon (26 patterns)

The positive lexicon includes patterns for general positive adjectives, expressions of satisfaction, perceived utility, and learning outcomes. Morphological variants are captured via regex alternations.

#### Negative Lexicon (28 patterns)

The negative lexicon includes general negative adjectives, emotional responses, technical complaints, excess indicators, and negated positive terms.

#### Negation Handling

A dedicated set of 10 negation markers was used to detect cases where positive terms are negated in context. When a negation marker appeared in proximity to a positive root term, the positive count was decremented by one and the negative count was incremented by one. This handles common constructions that would otherwise be misclassified as positive.

#### Classification Rules

For each comment, the number of positive matches (n_pos) and negative matches (n_neg) were counted after negation adjustment. The final sentiment label was assigned as follows:

| **Label** | **Condition** | **Interpretation** |
| --- | --- | --- |
| Positive | n_pos > 0 and n_neg = 0 | Only positive terms detected |
| Negative | n_neg > 0 and n_pos = 0 | Only negative terms detected |
| Mixed_Positive | n_pos > 0 and n_neg > 0 and ratio > 0.6 | Both present, predominantly positive |
| Mixed_Balanced | n_pos > 0 and n_neg > 0 and 0.4 ≤ ratio ≤ 0.6 | Both present, balanced |
| Mixed_Negative | n_pos > 0 and n_neg > 0 and ratio < 0.4 | Both present, predominantly negative |
| Neutral | n_pos = 0 and n_neg = 0 | No evaluative terms detected |

Note: ratio = n_pos / (n_pos + n_neg). For simplified analyses (e.g., boxplots), Mixed_Positive, Mixed_Negative, and Mixed_Balanced were collapsed into a single “Mixed” category.

### Thematic Coding

Thematic content was identified using 11 predefined dictionaries, each consisting of domain-specific Spanish-language regex patterns. Multiple themes could be assigned to a single comment. The primary theme was determined by the dictionary with the highest number of pattern matches within the comment.

| **Theme** | **# Patterns** | **Description** |
| --- | --- | --- |
| UTILITY | 15 | Perceived usefulness for studying, revision, exam preparation |
| ERRORS | 14 | Content errors, incorrect answers, labeling mistakes |
| SAVE_ISSUE | 13 | Save/progress loss, platform data issues |
| LENGTH | 11 | Excessive length, workload, time demands |
| IMAGES | 11 | Image quality, clarity, labeling of visual elements |
| FORMAT | 11 | Numbering, ordering, structural issues |
| LEARNING | 10 | Learning, educational, formative value |
| TIME | 11 | Time investment, time loss |
| PLATFORM | 11 | Technical failures, bugs, crashes |
| DYNAMIC | 7 | Engagement, interactivity, enjoyment |
| VISUAL | 6 | Visual learning, spatial identification |

Note: All patterns use regex with morphological variants (gender, number, verb conjugations) to maximize recall. Comments with no theme matches were classified as OTHER.

### NPS–Comment Coherence Validation

To assess the internal consistency between quantitative (NPS score) and qualitative (comment sentiment) responses, a coherence analysis was performed. Each response was classified as Coherent or Incoherent based on the following rules:

| **NPS Category** | **Comment Sentiment** | **Classification** |
| --- | --- | --- |
| Promoter (NPS 9–10) | Positive, Mixed_Positive, Mixed_Balanced, or Neutral | Coherent |
| Promoter (NPS 9–10) | Negative or Mixed_Negative | Incoherent |
| Passive (NPS 7–8) | Any sentiment | Coherent (by definition) |
| Detractor (NPS 0–6) | Negative, Mixed_Negative, Mixed_Balanced, or Neutral | Coherent |
| Detractor (NPS 0–6) | Positive or Mixed_Positive | Incoherent |

Additionally, five specific incoherence types were defined to identify the most discrepant cases:

| **Type** | **Definition** |
| --- | --- |
| T1 | Promoter (9–10) with Negative or Mixed_Negative sentiment |
| T2 | Detractor (0–6) with purely Positive sentiment |
| T3 | NPS = 10 with ≥ 2 negative words |
| T4 | NPS ≤ 4 with Positive or Mixed_Positive sentiment |
| T5 | NPS ≥ 9 with more negative than positive words and mentions of ERRORS, SAVE_ISSUE, or PLATFORM themes |

Incoherent cases were individually reviewed to verify the classification. The detailed list of all incoherent cases, including their original comments, NPS scores, and classified sentiment, is provided in Supplementary Table S5.

### Statistical Testing of Sentiment–NPS Association

To test whether NPS scores differed across sentiment categories, a Kruskal–Wallis H test was conducted using the simplified four-level sentiment variable (Positive, Mixed, Negative, Neutral) as the grouping factor and NPS score (0–10) as the dependent variable. The Kruskal–Wallis test is a non-parametric rank-based test appropriate for comparing the distributions of a continuous variable across three or more independent groups when the normality assumption is violated. The test statistic H is calculated as:

H = [12 / N(N+1)] × Σ(Rᵢ² / nᵢ) − 3(N+1)

where N is the total sample size, ni is the size of group i, and Ri is the sum of ranks in group i. Under the null hypothesis that all groups come from the same distribution, H follows a chi-squared distribution with k − 1 degrees of freedom, where k is the number of groups. The resulting p-value is reported in Figure 3.

### Limitations

This content-based approach has several limitations that should be acknowledged:

Rule-based classification: The lexicons were manually constructed for this specific educational context. They may not generalize to other populations, languages, or feedback instruments. No external validation corpus was used.

No inter-rater reliability: Classification was performed algorithmically with manual review of discrepant cases, but no independent human coding was conducted for comparison. The coherence analysis (Supplementary Table S5) serves as a partial internal validation.

Single open-ended question: The qualitative component relied on a single prompt, which may constrain the depth and variability of responses compared to structured qualitative methodologies.

Context sensitivity: Short comments (e.g., “Good”, “too much”) may lack sufficient context for accurate sentiment classification. The minimum comment length was 4 characters.

Despite these limitations, the approach provides a transparent, reproducible, and fully documented method for extracting descriptive patterns from student feedback. The complete R code, including all lexicons and classification functions, is available in the script 03_phase3_response_patterns.R.
